# Prevalence and Characteristics of Needle Stick and Sharp Injuries Among King Salman Armed Forces Hospital Personnel in Tabuk City: A Retrospective Hospital-Based Study

**DOI:** 10.7759/cureus.43692

**Published:** 2023-08-18

**Authors:** Norah Alshehri, Salem Aldhahi, Abdulateef Sayed Elbadawi, Wareef Allahim, Aydah Alhowiti, Khalid Hassan, Walaa Abdelgwad

**Affiliations:** 1 Preventive Medicine, King Salman Armed Forces Hospital, Tabuk, SAU; 2 Family Medicine, Ministry of Health, Tabuk, SAU; 3 Preventive Medicine and Public Health, Ministry of Health, Tabuk, SAU; 4 Family Medicine, King Salman Armed Forces Hospital, Tabuk, SAU

**Keywords:** needle stick and sharp injuries, human immunodeficiency virus infection, who- world health organization, hepatitis b virus, healthcare workers

## Abstract

Background: This study aimed to determine the prevalence and associated characteristics of needle stick and sharp injuries (NSSIs) among King Salman Armed Forces Hospital (KSAFH) personnel.

Methods: Data was collected by reviewing all reported NSSIs among KSAFH personnel between January 2020 to December 2022.

Results: The prevalence of NSSIs was 2.05%, with nurses being the most commonly affected. Most injuries occurred in patients' rooms/wards and among health care workers (HCWs) aged < 40 years. Of the injured workers, 93.8% were immunized against hepatitis B virus (HBV).

Conclusion: Educational and training programs targeting high-risk age groups and professions should be developed. Policies related to locations and situations where injuries occur frequently should be reviewed regularly to reduce the risk of NSSIs.

## Introduction

The use of sharp materials by healthcare workers (HCWs) can result in needle stick and sharp injuries (NSSIs). These materials can puncture the skin when used during the screening, diagnosing, treating, or monitoring of a patient’s condition [1]. NSSIs include needles, cannulas, glass items, blades, lancets, scissors, pins, clamps, cutters, retractors, staples, and scalpels [2]. Typically, NSSIs cause minor bleeding or visible trauma to the skin surface and subcutaneous tissue. However, there is a risk of viral infection associated with NSSIs [3].

The nature of HCWs’ roles puts them at risk of sustaining accidental NSSIs. Almost 3 million HCWs sustain NSSIs and/or sharps injuries each year [4]. NSSIs continue to happen despite the World Health Organization's (WHO) introduction of guidelines for decreasing them within healthcare settings [5]. It is estimated that 32.4-44.5% of HCWs report at least one accidental NSSI each year [6]. In the USA, there are an estimated 385,000 annual NSSIs among HCWs, while in Europe, there are 1,000,000 reported annual NSSI cases among hospital HCWs [4].

NSSIs may be a major source of blood-borne disease infection, including hepatitis B (HBV), hepatitis C (HCV), and human immunodeficiency virus (HIV) [7]. The risk of transmission following a percutaneous injury with infected blood is 2-40% for HBV, 2.7-10% for HCV, and 0.3% for HIV [8]. Research indicates a link between injury and HCWs’ mental health. The quality of life of HCWs is negatively impacted by anxiety, sadness, and concern about transmitting infection to their families [9-10].

To lessen the risk of sustaining NSSIs among HCWs, preventive measures must be utilized by healthcare organizations. According to a meta-analysis, providing HCWs with engineered safety devices and training them on general safety precautions, safe injection techniques, and sharps waste disposal reduces NSSI occurrence by 62% [11]. According to UK guidelines, a robust injury reporting system and early post-exposure prophylactic use lower the risk of HIV infection [12].

The types of needles and sharp devices used, as well as their safety protocols, impact the risk of NSSIs among HCWs. The prevalence of NSSIs at healthcare institutions is also influenced by patient volume and the safety procedures that staff members perform when caring for them [13]. Screening, diagnosing, treating, and monitoring patients and managing medical waste presents a high risk of sustaining NSSIs to doctors, nurses, laboratory technicians, and medical waste management personnel [14]. HCWs may be more susceptible to NSSIs due to their stressful psychosocial working conditions and negative stress perception. Stressful psychosocial working conditions can increase health risks, which can be mitigated by managing stress perception [15].

Heavy workloads, working in operating rooms or intensive care units, having little work experience, and being young are all associated with NSSI incidence [16]. Unfortunately, most existing research on the connection between psychosocial work conditions and NSSIs has primarily treated these injuries as a stressor of psychosocial workplace conditions, ignoring the impact of stressful workplace conditions on their high incidence [17-18]. Furthermore, few studies have assessed psychosocial workplace circumstances as a contributor to acute injuries. Night shift work may greatly increase the risk of HCWs sustaining NSSIs [19]. HCWs report high levels of job stress, burnout, and discontent, which increases their risk of sustaining NSSIs [20].

A 2012 study involving 52 hospitals found that the national rate of NSSIs in Saudi Arabia was 3.2 per 100 occupied hospital beds [21]. According to data from King Saud Medical City in Riyadh, in 2009, there were 13.8 NSSIs for every 100 HCWs [22]. Other healthcare institutions in Saudi Arabia have reported varying rates [23-27]. However, these rates may be understated as injuries are occasionally not recorded. A review of studies on injury rates in the UK found that the estimated and reported rates varied by 10- to 100-fold [28]. While studies have sought to determine the prevalence and incidence of NSSIs among HCWs in Saudi Arabia, many have either focused on a small group of HCWs or only included regional universities [29-34].

To the best of our knowledge, no prior research has evaluated the incidence, prevalence, or characteristics of NSSIs among different medical professionals in the Tabuk region of Saudi Arabia. This investigation assessed the prevalence and characteristics of NSSIs among HCWs at King Salman Armed Forces Hospital (KSAFH) with diverse specialties between 2020-2022.

## Materials and methods

This hospital-based retrospective study was conducted at KSAFH in Tabuk City. KSAFH is a Joint Commission International-accredited hospital and provides tertiary healthcare services to military personnel and their eligible dependents. It has a capacity of 900 beds and has multiple linked primary care centers covering most of Tabuk City. The capital of Saudi Arabia's northwest is Tabuk, and its population in 2021 was approximately 667,000 [35]. The study population was HCWS at KSAFH, who were divided into medical and non-medical staff. Medical staff included physicians, nurses, lab technicians, clinical pharmacists, dentists, and dental assistants. Non-medical staff included housekeepers and laundry workers. We conducted a retrospective analysis of all reported cases of NSSI at KSAFH during the period from January 2020 to December 2022. All NSSI reports were referred by the infection control division to the employee health department and these reports were retrospectively examined. Age, gender, job title, location, site of the injury, and technique or task that resulted in the injury were used to categorize and determine the prevalence of NSSIs. A designated checklist was used for data collection which was adapted from the Exposure Prevention Information Network report, which provides standardized fields to record, monitor and analyze NSSIs and body fluid exposure events [36]. Data were statistically described in terms of frequencies (number of cases) and percentages. Because the sample size is small, a comparison between the study groups was made using Chi-square (c2) test based on Monte Carlo Exact test. Two-sided p-values less than 0.05 were considered statistically significant. IBM SPSS Statistics for Windows, Version 25 (IBM Corp, Armonk, NY, USA) was used for all statistical analyses. The KSAFH Research Ethical Committee granted ethical approval ethics ID number KSAFH-REC-2022-486. The confidentiality of participants’ personal information was ensured throughout the study by anonymizing the data.

## Results

Of the 2341 HCWs, housekeepers, and laundry workers included in this study, there were 48 NSSI cases, giving a prevalence of 2.05%. Over two-thirds of the reported injuries involved females (68.8%). Workers aged 39 years and younger comprised the majority of cases (66.6%). Of the three years evaluated, most occurred in 2021 (45.8%). More NSSIs occurred during the afternoon shift (39.6%). The critical care unit and housekeeping departments reported the highest numbers of injuries, each accounting for 18.8% of the total injuries. Nurses (41.6%) and housekeepers or laundry workers (25.0%) were the primary workers affected. Almost half of the reported injuries occurred in patients’ wards and rooms (47.8%). In most cases, the source patient was identifiable (72.9%), and the sharp item was contaminated (79.2%). In 56.3% of incidents, the victim was the original user of the sharp object. Injuries occurred primarily during sharp item use (33.3%) or after use but before disposal (33.3%). The hollow-bore needle was the main cause of injuries (70.8%). Injury depths were mostly moderate (52.1%) or superficial (43.8%). Most of the injured workers (93.8%) were immunized against HBV and were using a single pair of gloves (83.3%) (Table 1).

**Table 1:** Demographic characteristics and distribution of needle sticks and sharp injuries according to assessed variables of healthcare workers in KSAFH (N = 48) ER: emergency room HBV: hepatitis B virus KSAFH: King Salman Armed Forces Hospital

	Variable	N (%)
Gender	Male	15 (31.2)
	Female	33 (68.8)
Age group (years)	20-29	15 (31.2)
	30-39	17 (35.4)
	40-49	13 (27.1)
	> 49	3 (6.3)
Year of injury	2020	17 (35.4)
	2021	22 (45.8)
	2022	9 (18.8)
Time of injury	Morning shift (5 am-12 pm)	14 (29.2)
	Afternoon shift (12 pm-5 pm)	19 (39.6)
	Evening shift (5 pm-9 pm)	5 (10.4)
	Night shift (9 pm-4 am)	10 (20.8)
Employing department	Surgery	6 (12.5)
	Obstetrics and gynecology	5 (10.4)
	Internal medicine	2 (4.2)
	Pediatrics	1 (2.1)
	Critical care unit	9 (18.8)
	Dentistry	4 (8.3)
	ER	4 (8.3)
	Housekeeping	9 (18.8)
	Laundry	1 (2.1)
	Others	7 (14.6)
Job title	Physician	10 (20.8)
	Nurse	20 (41.6)
	Technician	2 (4.2)
	Dentist or dental assistant	4 (8.4)
	Housekeeper or laundry worker	12 (25.0)
Location where the injury occurred	Outpatient clinic or procedure room	6 (12.5)
	Patient room/ward	23 (47.8)
	Operating room/recovery or labor room	6 (12.5)
	Intensive/critical care unit or emergency department	7 (14.6)
	Laboratories or blood bank	3 (6.3)
	Other	3 (6.3)
Was the source patient identifiable?	Yes	35 (72.9)
	Unknown	13 (27.1)
Was the injured worker the original user of the sharp item?	Yes	27 (56.3)
	No	21 (43.7)
Was the sharp item contaminated?	Yes	38 (79.2)
	No	2 (4.2)
	Unknown	8 (16.7)
When did the injury occur?	During item use	16 (33.3)
	Between steps in a multi-step procedure	3 (6.3)
	After use but before item disposal	16 (33.3)
	During disposal	3 (6.3)
	After disposal	3 (6.3)
	Unknown	7 (14.6)
What device caused the injury?	Hollow-bore needle	34 (70.8)
	Surgical and solid needle	11 (22.9)
	Unknown	3 (6.3)
Degree of injury	Superficial (little or no bleeding)	21 (43.8)
	Moderate (skin punctured, some bleeding)	25 (52.1)
	Severe (deep cut or profuse bleeding)	2 (4.2)
What was penetrated by the sharp item?	Single pair of gloves	40 (83.3)
	Double pair of gloves	7 (14.6)
	No gloves	1 (2.1)
Was the injured worked immunized status against HBV?	Immunized	45 (93.8)
	Not immunized	2 (4.2)
	Unknown	1 (2.1)

The job title of the participants was compared to the other independent variables. In all comparisons, nurses comprised the majority of injured workers. The greater number (22.9%) of injuries among nurses occurred significantly (P=0.000*) in the patients' room/ward. The source of infection was significantly (P=0.000*) identifiable in the superiority (37.5%) of the injured nurses. The sharp item causing the injury was significantly (P=0.026*) contaminated (37.5%). The injury occurred significantly (P=0.003*) during the use of the sharp item (14.6%) or after its use and before its disposal (16.7%). The hollow-bore needle was significantly (P=0.000*) the most frequent device (37.5%) causing injury to the nurses. The injured nurses were significantly (P=0.000*) the original user of the sharp item in most cases (31.3%). The affected nurses were mainly females (35.4%), aged 30-39 Y (18.8%) in 2021 (22.9%) especially (16.7%) on the night shift (9 pm-4 am). The injury occurred in nurses more by contaminated items (37.5%). Most injured nurses were immunized (41.7%), using single pair of gloves (37.5%), and suffered a moderate injury (22.9%) (Table 2).

**Table 2:** Comparison of needle stick and sharp injuries by job title to independent variables of healthcare workers in KSAFH (N = 48) *Significant = (P-value is less than 0.05) KSAFH: King Salman Armed Forces Hospital

		Physician	Nurse	Technician	Dentist & dental assistant	Housekeeper & laundry worker	Total	P-value
		N (%)	N (%)	N (%)	N (%)	N (%)	N (%)	
Gender	Male	5 (10.4)	3 (6.3)	1 (2.1)	0 (0.0)	6 (12.5)	15 (31.3)	0.061
	Female	5 (10.4)	17 (35.4)	1 (2.1)	4 (8.3)	6 (12.5)	33 (68.7)	
Age group	20-29 Y	1 (2.1)	8 (16.7)	1 (2.1)	1 (2.1)	4 (8.3)	15 (31.3)	0.057
	30-39 Y	3 (6.3)	9 (18.8)	1 (2.1)	3 (6.3)	1 (2.1)	17 (35.4)	
	40-49 Y	4 (8.3)	3 (6.3)	0 (0.0)	0 (0.0)	6 (12.5)	13 (27.1)	
	> 49 Y	2 (4.2)	0 (0.0)	0 (0.0)	0 (0.0)	1 (2.1)	3 (6.3)	
Year of injury	2020	4 (8.3)	7 (14.6)	0 (0.0)	2 (4.2)	4 (8.3)	17 (35.4)	0.710
	2021	3 (6.3)	11 (22.9)	2 (4.2)	1 (2.1)	5 (10.4)	22 (45.8)	
	2022	3 (6.3)	2 (4.2)	0 (0.0)	1 (2.1)	3 (6.3)	9 (18.8)	
Time of injury	Morning shift (5 am-12 pm)	3 (6.3)	5 (10.4)	1 (2.1)	1 (2.1)	4 (8.3)	14 (29.2)	0.347
	Afternoon shift (12 pm-5 pm)	6 (12.5)	4 (8.3)	1 (2.1)	3 (6.3)	5 (10.4)	19 (39.6)	
	Evening shift (5 pm-9 pm)	0 (0.0)	3 (6.3)	0 (0.0)	0 (0.0)	2 (4.2)	5 (10.4)	
	Night shift (9 pm-4 am)	1 (2.1)	8 (16.7)	0 (0.0)	0 (0.0)	1 (2.1)	10 (20.8)	
Place of injury	Outpatient clinic & procedure room	3 (6.3)	0 (0.0)	0 (0.0)	3 (6.3)	0 (0.0)	6 (12.5)	0.000*
	Patients' room/ward	2 (4.2)	11 (22.9)	1 (2.1)	0 (0.0)	9 (18.8)	23 (47.9)	
	Operating room/Recovery (or labor room)	4 (8.3)	2 (4.2)	0 (0.0)	0 (0.0)	0 (0.0)	6 (12.5)	
	Intensive/Critical care unit (or emergency department)	1 (2.1)	5 (10.4)	0 (0.0)	0 (0.0)	1 (2.1)	7 (14.6)	
	Laboratories & blood bank	0 (0.0)	0 (0.0)	1 (2.1)	1 (2.1)	1 (2.1)	3 (6.3)	
	Others	0 (0.0)	2 (4.2)	0 (0.0)	0 (0.0)	1 (2.1)	3 (6.3)	
Was the source identifiable	Yes	10 (20.8)	18 (37.5)	1 (2.1)	3 (6.3)	3 (6.3)	35 (72.9)	0.000*
	Unknown	0 (0.0)	2 (4.2)	1 (2.1)	1 (2.1)	9 (18.8)	13 (27.1)	
Was the sharp item contaminated?	Uncontaminated	1 (2.1)	1 (2.1)	0 (0.0)	0 (0.0)	0 (0.0)	2 (4.2)	0.026*
	Contaminated	9 (18.8)	18 (37.5)	2 (4.2)	3 (6.3)	6 (12.5)	38 (79.2)	
	Unknown	0 (0.0)	1 (2.1)	0 (0.0)	1 (2.1)	6 (12.5)	8 (16.7)	
When did the injury occur	During the use of the item	7 (14.6)	7 (14.6)	0 (0.0)	2 (4.2)	0 (0.0)	16 (33.3)	0.003*
	Between steps of a multi-step procedure	2 (4.2)	1 (2.1)	0 (0.0)	0 (0.0)	0 (0.0)	3 (6.3)	
	After use, before disposal of the item	0 (0.0)	8 (16.7)	1 (2.1)	1 (2.1)	6 (12.5)	16 (33.3)	
	During disposal	0 (0.0)	2 (4.2)	0 (0.0)	0 (0.0)	1 (2.1)	3 (6.3)	
	After disposal	0 (0.0)	0 (0.0)	0 (0.0)	0 (0.0)	3 (6.3)	3 (6.3)	
	Unknown	1 (2.1)	2 (4.2)	1 (2.1)	1 (2.1)	2 (4.2)	7 (14.6)	
What type of device caused the injury?	Hollow-bore needle	5 (10.4)	18 (37.5)	1 (2.1)	0 (0.0)	10 (20.8)	34 (70.8)	0.000*
	Surgical and solid needle	5 (10.4)	2 (4.2)	0 (0.0)	4 (8.3)	0 (0.0)	11 (22.9)	
	Unknown	0 (0.0)	0 (0.0)	1 (2.1)	0 (0.0)	2 (4.2)	3 (6.3)	
Degree of the injury	Superficial	6 (12.5)	8 (16.7)	1 (2.1)	2 (4.2)	4 (8.3)	21 (43.8)	0.554
	Moderate	4 (8.3)	11 (22.9)	1 (2.1)	1 (2.1)	8 (16.7)	25 (52.1)	
	Severe	0 (0.0)	1 (2.1)	0 (0.0)	1 (2.1)	0 (0.0)	2 (4.2)	
Was the injured worker the original user of a sharp item?	Yes	8 (16.7%)	15 (31.3%)	1 (2.1)	3 (6.3)	0 (0.0)	27 (56.3%)	0.000*
	No	2 (4.2)	5 (10.4)	1 (2.1)	1 (2.1)	12 (25.0%)	21 (43.8%)	
What was penetrated by the sharp item (in hand injury)?	Single pair of gloves	8 (16.7)	18 (37.5)	1 (2.1)	4 (8.3)	9 (18.8)	40 (83.3)	0.369
	Double pair of gloves	2 (4.2)	1 (2.1)	1 (2.1)	0 (0.0)	3 (6.3)	7 (14.7)	
	No gloves	0 (0.0)	1 (2.1)	0 (0.0)	0 (0.0)	0 (0.0)	1 (2.1)	
Immunization status against HBV	Immunized	9 (18.8)	20 (41.7)	2 (4.2)	4 (8.3)	10 (20.8)	45 (93.8)	0.215
	Not immunized	0 (0.0)	0 (0.0)	0 (0.0)	0 (0.0)	2 (4.2)	2 (4.2)	
	Unknown	1 (2.1)	0 (0.0)	0 (0.0)	0 (0.0)	0 (0.0)	1 (2.1)	

Injury locations were compared against the other independent variables. In all comparisons, the patients' room/ward was the location where most injuries occurred. The greater number (22.9%) of the injured workers were significantly (P=0.000*) nurses. The causative device was significantly (P=0.000*) hollow-bore needle in the superiority (41.7%) of the injured nurses. Most injuries that occurred in this location occurred among females (37.5%) health workers, aged 20-29Y (18.8%), in the year 2021 (25.0%), particularly in the afternoon shift (16.7%) and the source of injury was identifiable (31.3%). The majority of injuries in the patients' room/ward occurred after the use and before disposal of the objects (18.8%), these objects caused moderate injury (29.2%). The injured workers were mostly immunized against HBV (43.8%) and used single pair of gloves (39.6%) (Table 3).

**Table 3:** Comparison of needle stick and sharp injuries by location of injury against the independent variables of healthcare workers in KSAFH (N = 48) *Significant = (P-value is less than 0.05) KSAFH: King Salman Armed Forces Hospital

		Outpatient clinic	Patients' room/ward	Operating room/Recovery	Intensive/Critical care unit	Laboratories & blood bank	Others	Total	P-value
		N (%)	N (%)	N (%)	N (%)	N (%)	N (%)	N (%)	
Gender	Male	1 (2.1)	5 (10.4)	3 (6.3)	3 (6.3)	1 (2.1)	2 (4.2)	15 (31.3)	0.429
	Female	5 (10.4)	18 (37.5)	3 (6.3)	4 (8.3)	2 (4.2)	1 (2.1)	33 (68.8)	
Age group	20-29 Y	1 (2.1)	9 (18.8)	1 (2.1)	2 (4.2)	1 (2.1)	1 (2.1)	15 (31.3)	0.217
	30-39 Y	4 (8.3)	5 (10.4)	1 (2.1)	4 (8.3)	2 (4.2)	1 (2.1)	17 (35.4)	
	40-49 Y	1 (2.1)	8 (16.7)	2 (4.2)	1 (2.1)	0 (0.0)	1 (2.1)	13 (27.1)	
	> 49 Y	0 (0.0)	1 (2.1)	2 (4.2)	0 (0.0)	0 (0.0)	0 (0.0)	3 (6.3)	
Year of injury	2020	2 (4.2)	5 (10.4)	2 (4.2)	4 (8.3)	1 (2.1)	3 (6.3)	17 (35.4)	0.071
	2021	1 (2.1)	12 (25.0)	4 (8.3)	3 (6.3)	2 (4.2)	0 (0.0)	22 (45.8)	
	2022	3 (6.3)	6 (12.5)	0 (0.0)	0 (0.0)	0 (0.0)	0 (0.0)	9 (18.8)	
Time of injury	Morning shift	3 (6.3)	6 (12.5)	2 (4.2)	0 (0.0)	1 (2.1)	2 (4.2)	14 (29.2)	0.162
	Afternoon shift	3 (6.3)	8 (16.7)	4 (8.3)	2 (4.2)	2 (4.2)	0 (0.0)	19 (39.6)	
	Evening shift	0 (0.0)	3 (6.3)	0 (0.0)	1 (2.1)	0 (0.0)	1 (2.1)	5 (10.4)	
	Night shift	0 (0.0)	6 (12.5)	0 (0.0)	4 (8.3)	0 (0.0)	0 (0.0)	10 (20.8)	
Job Title	Physician	3 (6.3)	2 (4.2)	4 (8.3)	1 (2.1)	0 (0.0)	0 (0.0)	10 (20.8)	0.000*
	Nurse	0 (0.0)	11 (22.9)	2 (4.2)	5 (10.4)	0 (0.0)	2 (4.2)	20 (41.7)	
	Technician	0 (0.0)	1 (2.1)	0 (0.0)	0 (0.0)	1 (2.1)	0 (0.0)	2 (4.2)	
	Dentist & dental assistant	3 (6.3)	0 (0.0)	0 (0.0)	0 (0.0)	1 (2.1)	0 (0.0)	4 (8.3)	
	Housekeeper & laundry worker	0 (0.0)	9 (18.8)	0 (0.0)	1 (2.1)	1 (2.1)	1 (2.1)	12 (25.0)	
Was the source identifiable	Yes	5 (10.4)	15 (31.3)	6 (12.5)	6 (12.5)	1 (2.1)	2 (4.2)	35 (72.9)	0.276
	Unknown	1 (2.1)	8 (16.7)	0 (0.0)	1 (2.1)	2 (4.2)	1 (2.1)	13 (27.1)	
Was the sharp item contaminated?	Uncontaminated	0 (0.0)	2 (4.2)	0 (0.0)	0 (0.0)	0 (0.0)	0 (0.0)	2 (4.2)	0.520
	Contaminated	5 (10.4)	15 (31.3)	6 (12.5)	7 (14.6)	3 (6.3)	2 (4.2)	38 (79.2)	
	Unknown	1 (2.1)	6 (12.5)	0 (0.0)	0 (0.0)	0 (0.0)	1 (2.1)	8 (16.7)	
When did the injury occur	During the use of the item	3 (6.3)	4 (8.3)	4 (8.3)	3 (6.3)	1 (2.1)	1 (2.1)	16 (33.3)	0.281
	Between steps of a multi-step procedure	1 (2.1)	0 (0.0)	2 (4.2)	0 (0.0)	0 (0.0)	0 (0.0)	3 (6.3)	
	After use, before disposal of the item	1 (2.1)	9 (18.8)	0 (0.0)	3 (6.3)	2 (4.2)	1 (2.1)	16 (33.3)	
	During disposal	0 (0.0)	2 (4.2)	0 (0.0)	1 (2.1)	0 (0.0)	0 (0.0)	3 (6.3)	
	After disposal	0 (0.0)	3 (6.3)	0 (0.0)	0 (0.0)	0 (0.0)	0 (0.0)	3 (6.3)	
	Unknown	1 (2.1)	5 (10.4)	0 (0.0)	0 (0.0)	0 (0.0)	1 (2.1)	7 (14.6)	
What type of device caused the injury?	Hollow-bore needle	1 (2.1)	20 (41.7)	2 (4.2)	7 (14.6)	2 (4.2)	2 (4.2)	34 (70.8)	0.000*
	Surgical & solid needle	5 (10.4)	0 (0.0)	4 (8.3)	0 (0.0)	1 (2.1)	1 (2.1)	11 (22.9)	
	Unknown	0 (0.0)	3 (6.3)	0 (0.0)	0 (0.0)	0 (0.0)	0 (0.0)	3 (6.3)	
Degree of the injury	Superficial	4 (8.3)	8 (16.7)	5 (10.4)	3 (6.3)	0 (0.0)	1 (2.1)	21 (43.8)	0.224
	Moderate	1 (2.1)	14 (29.2)	1 (2.1)	4 (8.3)	3 (6.3)	2 (4.2)	25 (52.1)	
	Severe	1 (2.1)	1 (2.1)	0 (0.0)	0 (0.0)	0 (0.0)	0 (0.0)	2 (4.2)	
Was the injured worker the original user of the sharp item?	Yes	4 (8.3)	11 (22.9%)	4 (8.3)	6 (12.5)	1 (2.1)	1 (2.1)	27 (56.3%)	0.387
	No	2 (4.2)	12 (25.0%)	2 (4.2)	1 (2.1)	2 (4.2)	2 (4.2)	21 (43.8%)	
What was penetrated by the sharp item)?	Single pair of gloves	6 (12.5)	19 (39.6)	5 (10.4)	7 (14.6)	2 (4.2)	1 (2.1)	40 (83.3)	0.342
	Double pair of gloves	0 (0.0)	3 (6.3)	1 (2.1)	0 (0.0)	1 (2.1)	2 (4.2)	7 (14.6)	
	No gloves	0 (0.0)	1 (2.1)	0 (0.0)	0 (0.0)	0 (0.0)	0 (0.0)	1 (2.1)	
Immunization status against HBV	Immunized	6 (12.5)	21 (43.8)	5 (10.4)	7 (14.6)	3 (6.3)	3 (6.3)	45 (93.8)	0.463
	Not immunized	0 (0.0)	2 (4.2)	0 (0.0)	0 (0.0)	0 (0.0)	0 (0.0)	2 (4.2)	
	Unknown	0 (0.0)	0 (0.0)	1 (2.1)	0 (0.0)	0 (0.0)	0 (0.0)	1 (2.1)	

## Discussion

NSSIs are a significant occupational hazard faced by HCWs globally. These injuries occur when a sharp instrument, such as a needle, scalpel, or broken glass, contaminated with blood from a source patient, accidentally punctures the skin. NSSIs have the potential to expose HCWs to a number of bloodborne infections, such as HBV, HCV, and HIV. NSIs not only pose a serious health risk to HCWs but have far-reaching implications for patient safety and healthcare delivery. According to WHO, NSSIs affect millions of HCWs globally each year. A systematic review and meta-analysis conducted by Bouya et al. estimated that the one-year global pooled prevalence of NSIs among HCWs was 44.5% [4]. Healthcare workers worldwide are projected to get 66000 HBV, 16000 HCV, and 200-5000 HIV infections annually as a result of sharps injuries. Globally, the proportions of HCWs who were exposed to HBV, HCV, and HIV through percutaneous occupational exposure were 37%, 39%, and 4.4%, respectively. Serious implications of these blood-borne diseases include long-term illness, disability, and death. [7].

Saudi Arabia has a rapidly growing healthcare sector and is, therefore, no exception to this issue. The epidemiology of NSSIs in Saudi Arabia has been a topic of concern among HCWs. Several studies have explored this using different methodologies. In our study, of the 2341 HCWs, housekeepers, and laundry workers included, 48 NSSIs were reported between January 2020 to December 2022, with a prevalence of 2.05% and peak occurrence in 2021. This prevalence is considered low in comparison with global and local published literature. Globally, an estimated 32.4-44.5% of HCWs report at least one accidental NSSI each year [4,6]. Memish et al. reported that 48% of HCWs in Saudi Arabia experience at least one NSSI in their career [37]. Another study by Abalkhail revealed that the annual incidence rate among HCWs in Saudi Arabia of NSIs estimated at 22.2% [38]. This difference may be due to variations in study designs, as many studies have used self-reported surveys [38-40]. while others have used retrospective analyses of NSSI reports [24,41-42].

One notable finding in our current study was the high prevalence of injuries among nurses, who accounted for 41.6% of all injured workers. This aligns with previous research highlighting the increased risk of NSSIs among nurses due to their frequent and direct patient contact. Moreover, a higher proportion of injuries occurred in female HCWs (68. 8%), which may be attributed to their higher representation in nursing roles. This result lines up with research from related investigations undertaken in Saudi Arabia, Turkey, Indonesia, Italy, and Kenya. [2,5,43-45].

Patient rooms/wards were the most common place of NSSI occurrence, followed by critical care units. This is not surprising and has been reported in previous studies, as these areas are where HCWs spend a significant amount of time providing direct care [21,46]. This finding underscores the importance of implementing safety protocols and measures within these settings to minimize injury risk. Injuries sustained in these areas primarily occurred in the 20-29-year-old age group suggesting that early-career HCWs may be particularly vulnerable to these types of injuries. This may be due to a lack of experience, insufficient training, or inadequate adherence to safety protocols [47].

In the current study, most injuries occurred during item use or after use but before disposal, and most were caused by hollow-bore needles. Furthermore, in most cases, the source patient was identifiable, and the sharp item was contaminated. A systematic review and meta-analysis of the incidence and etiology of NSSIs worldwide demonstrated a similar finding [48]. This may imply a potential gap in HCWs’ adherence to safe disposal practices and is consistent with other previous studies that have emphasized the need for comprehensive training programs addressing safe use and proper disposal practices to minimize the risk of injuries and potential exposure to infectious materials [49-50].

Fortunately, most of the injured workers were immunized against HBV and had used a single pair of gloves. The high rate of immunization indicates positive adherence to vaccination protocols. This finding conforms with previous research that has emphasized the importance of immunization in protecting HCWs against blood-borne pathogens [47]. Ongoing education and awareness regarding the importance of immunization and regular vaccination updates should be provided to maintain optimal protection. This study has limitations related to data completeness, which may have resulted in the prevalence of NSSIs being underestimated. This may be due to HCWs underreporting due to a lot of causes like fear of reporting may affect their career, belief regarding the risk of infection transmission will be very low, a lack of knowledge toward the benefits and importance of post-exposure prophylaxis, and time-consuming reporting methods. As a result of this and the fact that all of the participants were drawn from a single institution, the findings' generalizability may be restricted due to the small sample size.

## Conclusions

The aim of this study was to identify the prevalence of occupational NSSI exposure and its associated characteristics. We reported an NSSI prevalence of 2.05% among HCWs at KSAFH, Tabuk City. Nurses were the most commonly affected, followed by housekeepers and physicians. In addition, NSSIs were more common among workers aged < 40 years, and most occurred in patient rooms/wards and critical care units. Regarding immunization status, most injured workers were immunized against HBV. We advise creating an educational and mentoring program, targeting high-risk age groups and professions. Regularly reviewing policies related to locations and situations where NSSIs occur frequently could help increase awareness and employee adherence to safe sharps practices and disposal. Hospitals should provide and enforce the use of engineering controls to reduce the future risk of NSSIs. A further study that observe unsafe practices is recommended in the future.
